# Supplementary material for: Adaptive responses of marine diatoms to zinc scarcity and ecological implications
Source: Nat Commun. 2022 Apr 14;13:1995. doi: 10.1038/s41467-022-29603-y (PMC9010474; doi:10.1038/s41467-022-29603-y)
Supplement: Supplementary file 1 — Supplementary Information [file 41467_2022_29603_MOESM1_ESM.pdf]

Supplementary information for Adaptive Responses of Marine Diatoms to Zinc Scarcity and Ecological Implications

Supplementary Discussion

Detection of ZCRP-A in Prochlorococcus

While detected ZCRP-B homologs (with >30% identity) were restricted to eukaryotic species, ZCRP-A homologs were not. We note that proteins identified from 25 unique contigs from the METZYME expedition had significant similarity (>30% identity, E $<10^{-10}$) to ZCRP-A and had taxonomic best matches to the cyanobacteria *Prochlorococcus marinus*. Prokaryotes are known to have minimal requirements for—and even toxicity to— Zn when grown in culture[2,68,69], have fewer overall Zn binding domains in their genomes compared to eukaryotes[70], and have absolute requirements for Co that cannot be met by Zn (Sunda and Huntsman 1995; Saito et al. 2002, 2003). As described previously, all COG0523 proteins are so classified by the presence of conserved binding domains (Fig. 3), though even homologs within the subclass have different functions and metal binding affinities[19]. The ZCRP-A homolog detected in *Prochlorococcus* is therefore more likely to be a true CobW protein with a role in Co metabolism, namely in the production of the cyanobacterial type of vitamin B_{12} pseudocobalamin[71]. Recent work has described how mis-metalation of CobW with Zn^{2+} is thermodynamically possible[19], which suggests a mechanism to explain the observed sensitivity of Co-limited *Prochlorococcus* to Zn toxicity[69]. In addition to the role of COG0523 proteins in *Prochlorococcus,* the potential for Cd^{2+} binding or a response to Cd^{2+} warrants future investigation as cambialistic metabolic cycling of Zn, Co and Cd are related in marine phytoplankton.

Supplementary Table 1. Growth rates and average spectral counts of ZCRP-A and ZCRP-B detected in diatom cultures used in this study. Growth experiments were conducted in biological duplicate (replicates A and B). Metal concentrations are provided as the amount of metal added to each media treatment ("Added [metal]" columns) and the concentration of free metal ions present in each treatment ("[metal^{2+}]" columns). Free metal ion concentrations were calculated using the total metal concentration (background media concentration + added concentration) and media composition based on equilibration with 10^{-4} M EDTA. NA, data not available; ND, not detected. Spectral counts are given as the average of technical triplicate measurements for Zn treatments of *T. pseudonana* and all *P. tricornutum* data and as single measurements for Co treatments of *T. pseudonana*, *P. delicatissima*, and *Chaetoceros* RS19.

Diatom	Added [Zn] (nmol L^{-1})	Added [Co] (nmol L^{-1})	[Zn^{2+}] (pmol L^{-1})	[Co^{2+}] (pmol L^{-1})	Growth rate replicate A	Growth rate replicate B	Growth rate average (d^{-1})	ZCRP-A spectral counts	ZCRP-B spectral counts
T. pseudonana	0	0	0.09	0.02	0	0	0	NA	NA
T. pseudonana	0	1	0.09	0.26	0.13	0.23	0.18	NA	NA
T. pseudonana	0	3	0.09	0.72	0.23	0.3	0.27	NA	NA
T. pseudonana	0	10	0.09	2.34	0.38	0.38	0.38	42	267
T. pseudonana	0	30	0.09	7.08	0.38	0.32	0.35	14	102
T. pseudonana	0	100	0.09	23.4	0.68	0.66	0.67	10	64
T. pseudonana	1	0	0.19	0.02	0.23	ND	0.23	NA	NA
T. pseudonana	3	0	0.40	0.02	0.28	0.37	0.33	NA	NA
T. pseudonana	10	0	1.12	0.02	0.57	0.65	0.61	34	109
T. pseudonana	30	0	3.16	0.02	0.69	0.74	0.72	30	105
T. pseudonana	100	0	10.23	0.02	0.65	0.72	0.69	ND	ND
P. tricornutum	0	0.1	0.09	0.05	0.56	0.45	0.51	226	67
P. tricornutum	0	3	0.09	0.72	0.48	0.52	0.5	77	21
P. tricornutum	0	10	0.09	2.34	0.61	0.58	0.6	85	15
P. tricornutum	0	30	0.09	7.08	0.62	0.59	0.61	22	10
P. tricornutum	0	100	0.09	23.4	0.71	NA	0.71	ND	ND
P. tricornutum	0.1	0	0.10	0.02	0.60	0.63	0.62	192	16
P. tricornutum	1	0	0.19	0.02	0.61	0.73	0.67	67	9
P. tricornutum	3	0	0.40	0.02	0.67	0.67	0.67	42	17
P. tricornutum	10	0	1.12	0.02	0.79	0.82	0.81	1	ND
P. tricornutum	30	0	3.16	0.02	0.80	0.80	0.80	ND	ND
P. tricornutum	100	0	10.23	0.02	0.78	0.81	0.8	ND	ND
P. delicatissima	0	0	0.09	0.02	0.09	0.04	0.06	NA	NA
P. delicatissima	0	1	0.09	0.26	0.26	0.23	0.24	NA	NA
P. delicatissima	0	3	0.09	0.72	0.59	0.54	0.56	NA	NA
P. delicatissima	0	10	0.09	2.34	0.60	0.68	0.64	13	9
P. delicatissima	0	30	0.09	7.08	0.68	0.56	0.62	19	15
P. delicatissima	0	100	0.09	23.4	0.74	0.76	0.75	9	6
P. delicatissima	1	0	0.19	0.02	0.38	0.5	0.44	NA	NA
P. delicatissima	3	0	0.40	0.02	0.49	0.53	0.51	NA	NA
P. delicatissima	10	0	1.12	0.02	0.61	0.51	0.56	9	3
P. delicatissima	30	0	3.16	0.02	0.64	0.76	0.7	11	5
P. delicatissima	100	0	10.23	0.02	0.58	0.64	0.61	4	ND
Chaetoceros RS19	0	0	0.07	0.02	0.01	0.02	0.01	NA	NA
Chaetoceros RS19	0.1	0	0.08	0.02	0.03	0.02	0.03	NA	NA
Chaetoceros RS19	1	0	0.17	0.02	0.14	NA	0.14	NA	NA
Chaetoceros RS19	3	0	0.38	0.02	0.37	0.41	0.39	NA	NA
Chaetoceros RS19	10	0	1.12	0.02	0.39	0.37	0.38	49	ND
Chaetoceros RS19	30	0	3.16	0.02	0.43	0.43	0.43	10	ND
Chaetoceros RS19	100	0	10.23	0.02	0.38	0.49	0.44	6	ND

Supplementary Table 2. Proteins with significant sequence similarity to *P. tricornutum* ZCRP-A and ZCRP-B found in the METZYME 3µm metaproteomic database.

Protein hit	Organism (nearest taxonomic match)	METZYME 3µm Protein ID	Pfam description	E value	% Identity
ZCRP-A	*Azadinium spinosum* 3D9	contig_1613015_1_828_+	CobW/HypB/UreG, nucleotide-binding domain	4.6e-76	53.71
ZCRP-A	*Helicotheca tamensis* strain CCMP826	contig_1553126_100_849_-	CobW/HypB/UreG, nucleotide-binding domain	3.1e-103	60.73
ZCRP-A	*Emiliania huxleyi* CCMP1516	contig_1751519_1621_2010_+	CobW/HypB/UreG, nucleotide-binding domain	1.9e-63	77.31
ZCRP-A	*Phaeocystis sp.* CCMP2710	contig_1752308_1_806_+	CobW/HypB/UreG, nucleotide-binding domain	4.3e-54	43.11
ZCRP-B	*Pseudo-nitzschia fraudulenta* WWA7	contig_1892291_1_1034_+	Bacterial extracellular solute-binding proteins, family 5 Middle	1.2e-85	46.30
ZCRP-B	*Leptocylindrus danicus* B651	contig_1022443_1_1026_+	Bacterial extracellular solute-binding proteins, family 5 Middle	9.0e-64	40.94
ZCRP-B	*Azadinium spinosum* 3D9	contig_666973_1_713_-	Bacterial extracellular solute-binding proteins, family 5 Middle	1.6e-30	52.78
ZCRP-B	*Symbiodinium type D1a*	contig_1229636_874_1902_-	Bacterial extracellular solute-binding proteins, family 5 Middle	1.24e-94	50.00
ZCRP-B	*Gonyaulax spinifera, Strain CCMP409*	contig_1206767_1_1974_-	Bacterial extracellular solute-binding proteins, family 5 Middle	1.5e-130	45.68

Supplementary Table 3. IDs of ZCRP proteins described in this study.

Protein	Organism	Protein ID	NCBI Accession Number
ZCRP-A	*Thalassiosira pseudonana* CCMP1335	3054 (JGI Thaps3*)	XP_002287602.1
ZCRP-B	*Thalassiosira pseudonana* CCMP1335	938 (JGI Thaps3_bd**)	XP_002297477.1
ZCRP-A	*Phaeodactylum tricornutum* CCMP632	41508 (JGI Phatr2†)	XP_002185475.1
ZCRP-B	*Phaeodactylum tricornutum* CCMP632	49168 (JGI Phatr2†)	XP_002183815.1
ZCRP-A	*Pseudo-nitzschia delicatissima* UNC1205	CAMPEP_0197265366‡	NA
ZCRP-B	*Pseudo-nitzschia delicatissima* UNC1205	CAMPEP_0116079096‡	NA
ZCRP-A	*Chaetoceros sp.* RS19	contig_24506_685_2565_-§	NA

*Joint Genome Institute Thaps3 database (https://mycocosm.jgi.doe.gov/Thaps3/Thaps3.home.html)

**Joint Genome Institute Thaps3_bd database (https://mycocosm.jgi.doe.gov/Thaps3_bd/Thaps3_bd.home.html

† Joint Genome Institute CCAP 1055/1 v2.0 Phatr2, all models database (https://mycocosm.jgi.doe.gov/Phatr2/Phatr2.home.html)

‡ Protein ID from *P. delicatissima* transcriptome (transcriptome ID EP00533) available through the EukProt database [67].

§ *Chaetoceros* RS19 translated transcriptome database submitted to the ProteomeXchange Consortium through the PRIDE [65] partner repository with dataset identifier PXD026895

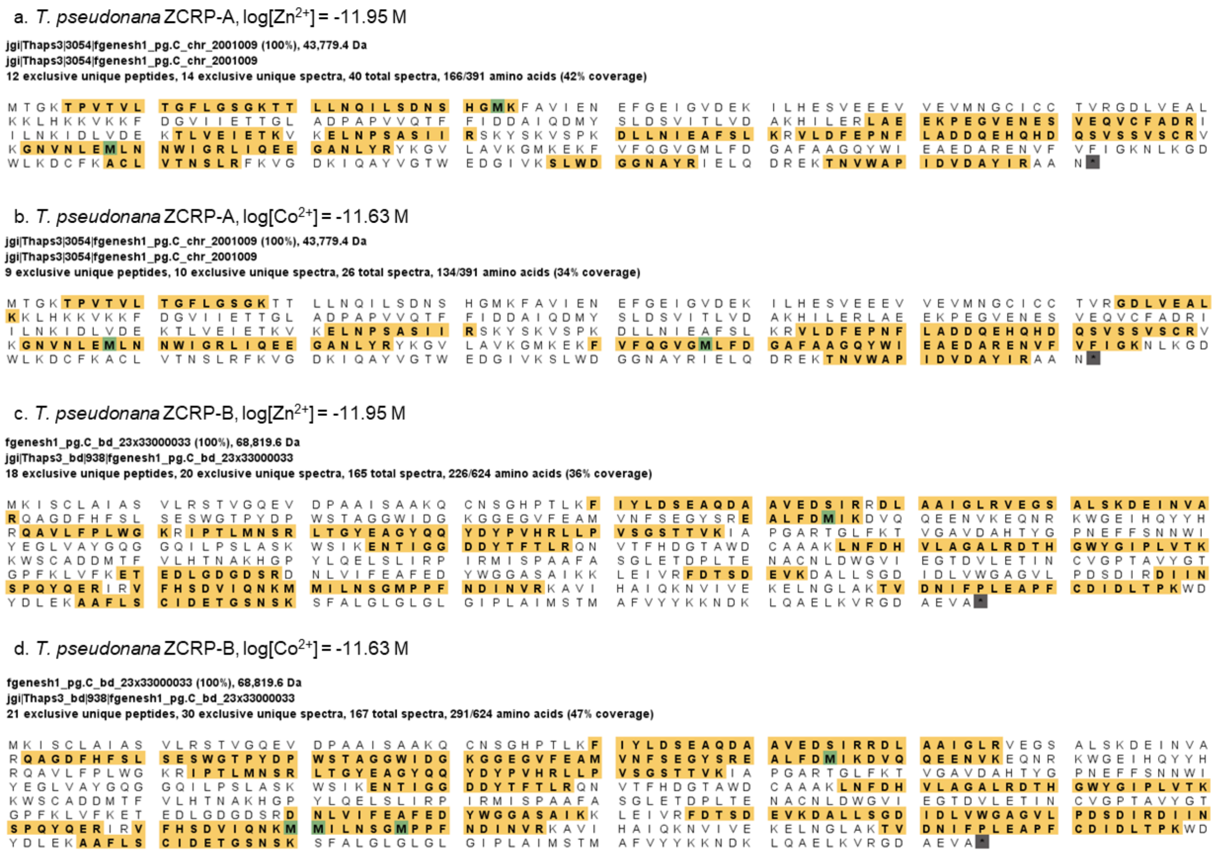

Supplementary Figure 1. Exclusive unique peptides identified for *Thalassiosira pseudonana* CCMP1335 ZCRP-A (a,b) and ZCRP-B (c,d) in low Zn^{2+} (log[Zn^{2+}] = -11.95 M) and low Co^{2+} (log[Co^{2+}] = -11.63 M) treatments visualized in Scaffold 5. The number of exclusive unique peptides that map to each protein sequence are as follows: (a) 12, (b) 9, (c) 18, and (d) 21. For both proteins in all cases, protein probabilities were 100%.

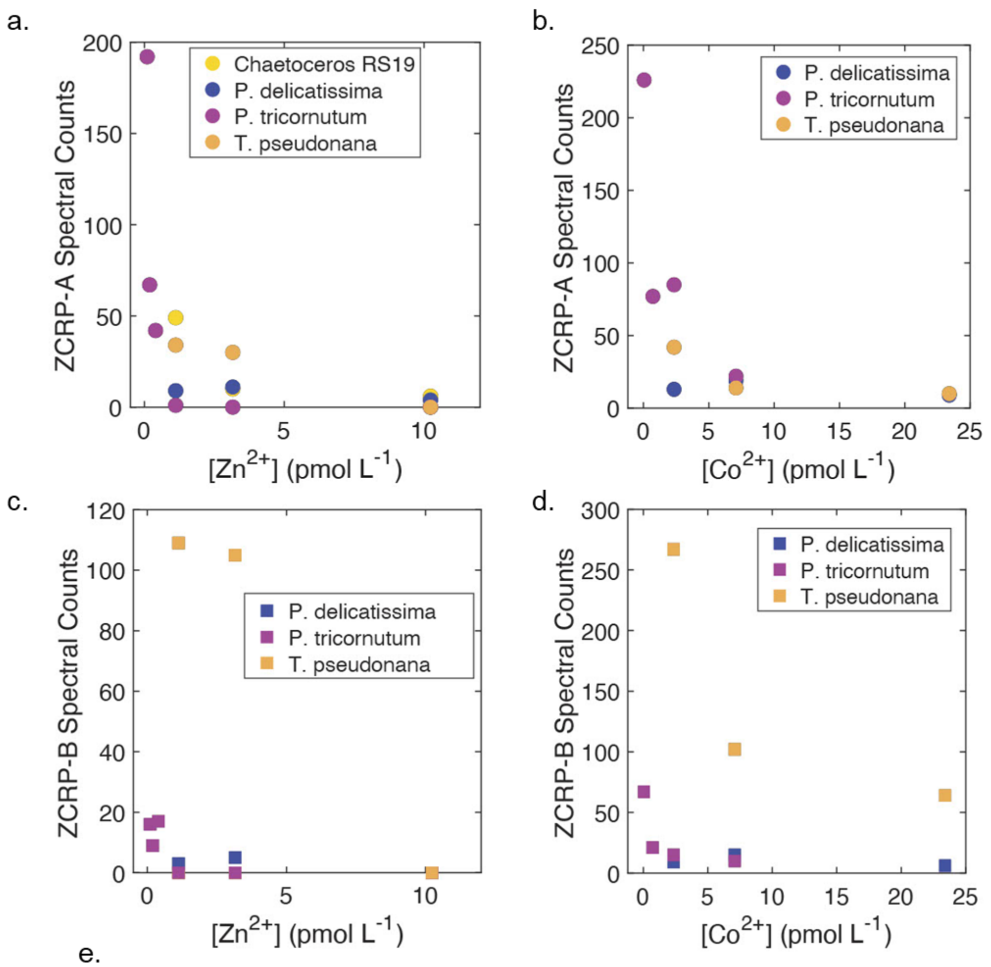

e.

	Zn		Co	
	τ	p	τ	p
ZCRP-A	-0.67	0.002	-0.73	0.007
ZCRP-B	-0.48	0.034	-0.27	0.353

Supplementary Figure 2.
Diatom culture data comparing ZCRP-A spectral counts (a,b) and ZCRP-B spectral counts (c,d) to total media divalent metal cation concentration. (e) Kendall correlation statistics showing correlation coefficients (tau) and significance (p) for correlations between ZCRPA/B spectral counts and Zn/Co concentrations. Data plotted from Table S1. N values used for statistical tests are as follows: (a) n = 15, (b) n = 11, (c), n = 12, (d) n = 11. To assess the significance of the relationship between metal concentration and ZCRP-A/B abundance, we utilized the Kendall-tau rank correlation test. This test does not assume *a priori* a relationship between two indices, and instead tests whether the ranked order of two quantities is significantly correlated. The correlation is two-tailed and can vary from -1 to 1, with -1 being perfectly anti-correlated, 0 being no relationship at all, and 1 being perfectly correlated.

a.

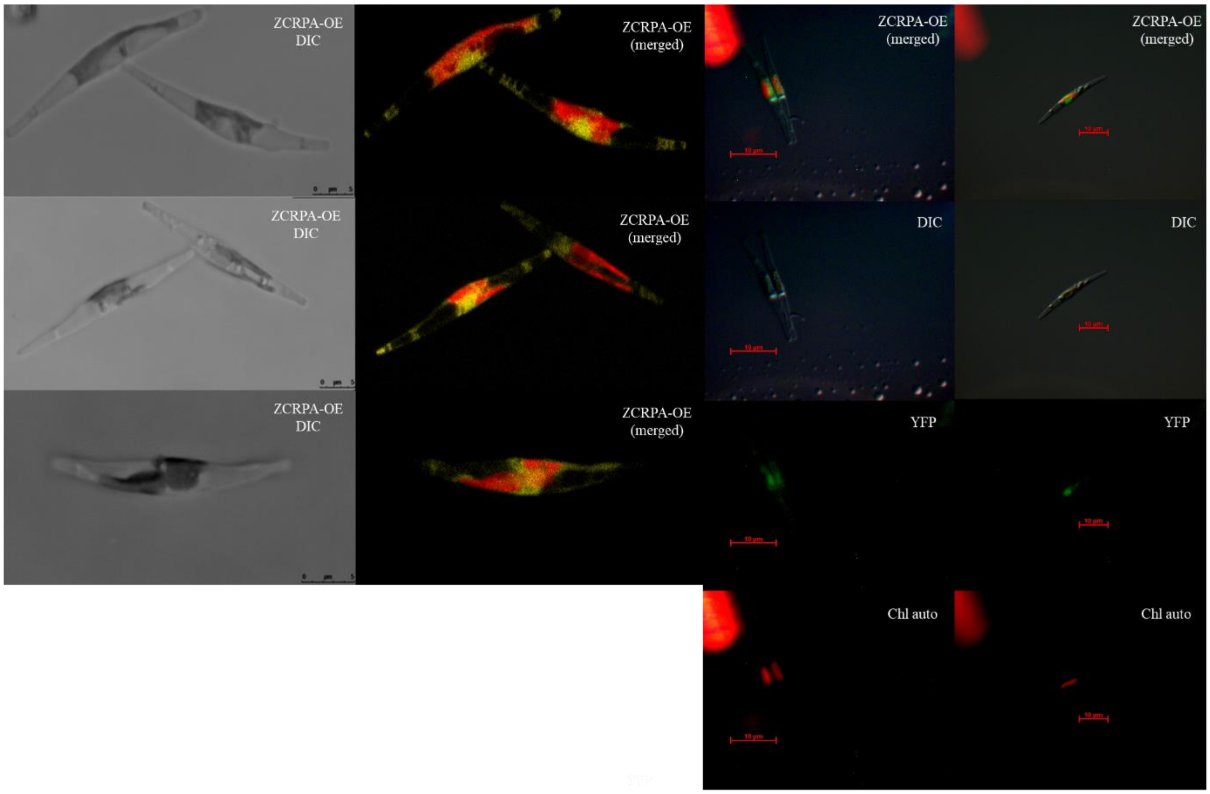
ZCRPA-OE
DIC
ZCRPA-OE
(merged)
ZCRPA-OE
(merged)
ZCRPA-OE
(merged)
ZCRPA-OE
DIC
ZCRPA-OE
(merged)
DIC
DIC
ZCRPA-OE
DIC
ZCRPA-OE
(merged)
YFP
YFP
Chl auto
Chl auto

b.

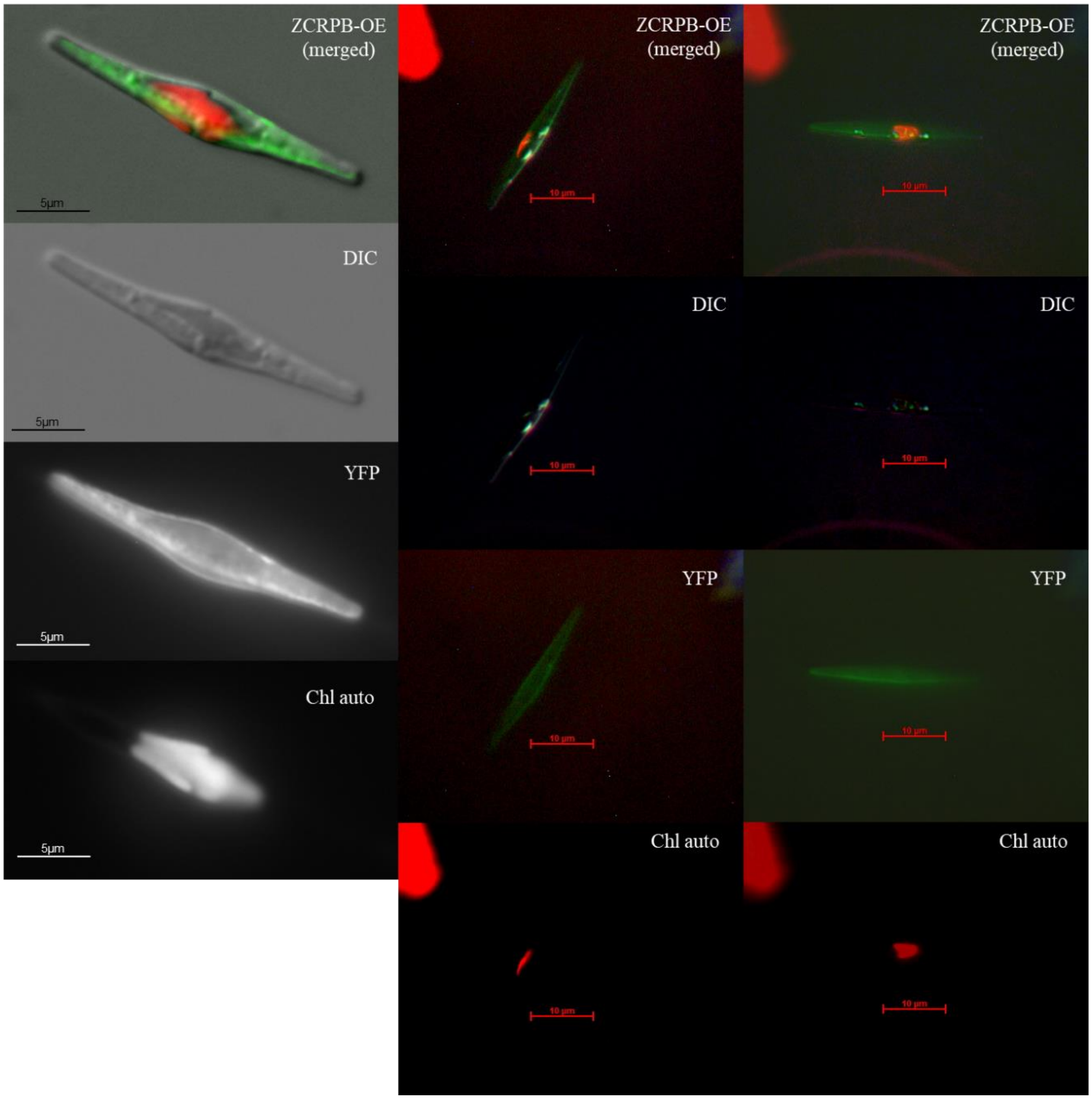

Supplementary Figure 3. Additional epifluorescent micrographs of (a) ZCRP-A and (b) ZCRP-B proteins fused to YFP and overexpressed in *P. tricornutum* (ZCRPA-OE and ZCRPB-OE, respectively) showing localization of ZCRP-A near the chloroplasts and ZCRP-B to the cell membrane. YFP fluorescence is false-colored green and chlorophyll autofluorescence is false-colored red. Composite (merged) images are stacks of the individual channels differential interference contrast (DIC), yellow fluorescent protein (YFP), and chlorophyll autofluorescence (Chl auto). Black scale bar in (a) is 5µm. Red scale bars are 10µm. Results were validated > 10 times.

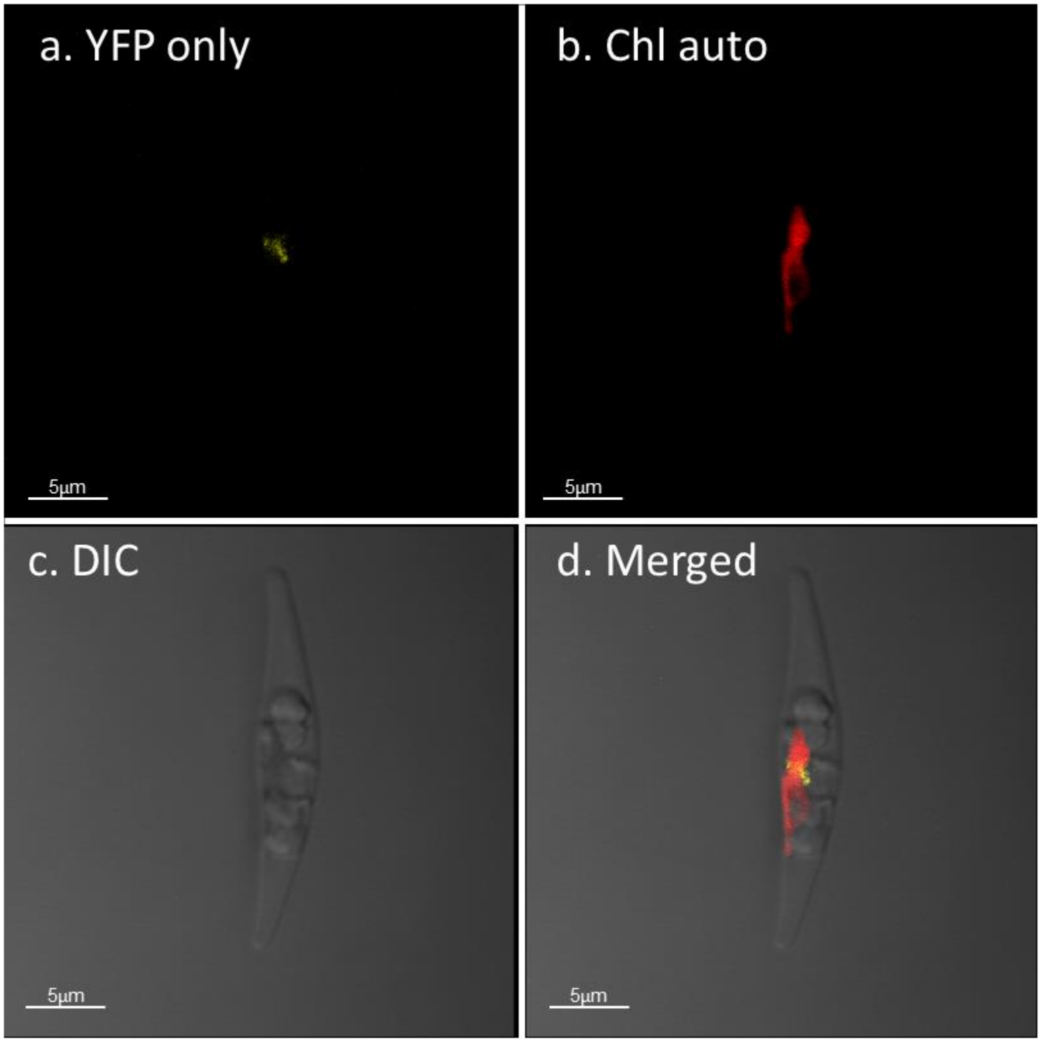

Supplementary Figure 4. Epifluorescent micrographs showing distribution of yellow fluorescent protein (YFP) alone (untagged to any protein) in a representative *P. tricornutum* cell. YFP fluorescence (a) is false-colored green and chlorophyll autofluorescence (b) is false-colored red. (c) Differential interference contrast (DIC) image. (d) Merged image comprised of the individual channels shown in (a), (b) and (c). Results were validated > 10 times.

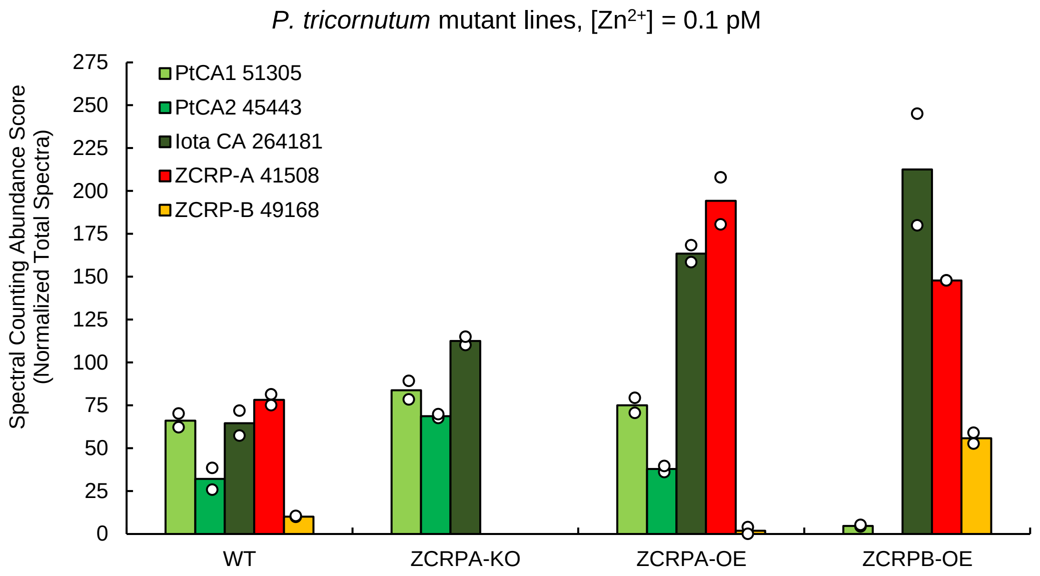

Supplementary Figure 5. Mean spectral counting abundance scores of CAs and ZCRPs detected in low [Zn^{2+}] (0.1pM) treatments of wild-type (WT), *ZCRPA*-knockout (KO), and *ZCRPA*-overexpression (OE) lines of *P. tricornutum* as measured by global proteomic analysis. Data is presented as mean values of technical duplicate measurements of pooled biological duplicates (n=2). Individual data points are overlaid as white circles. Protein names are shown with their corresponding JGI protein ID. ND, not detected.

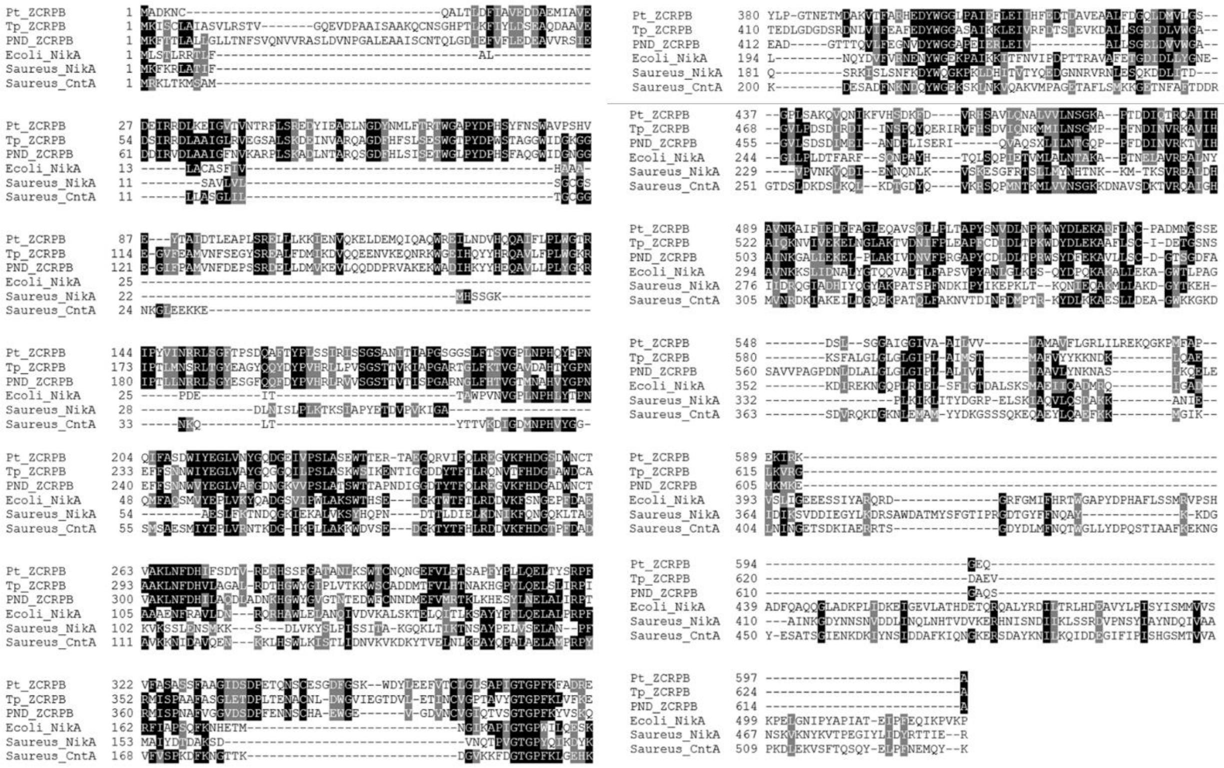

Supplementary Figure 6. Complete sequence alignment of *P. tricornutum* ZCRP-B compared to the ZCRP-B homologs in *T. pseudonana* (Tp) and *P. delicatissima* (PND) in addition to NikA in *E. coli*, NikA in *S. aureus,* and CntA in *S. aureus. P. tricornutum* ZCRP-B shares 41.1% identity with *T. pseudonana* ZCRP-B (E=2e-151), 41.3% identity with *P. delicatissima* ZCRP-B (E=3e-145), 30.5% identity with *E. coli* NikA (E=7e-49), 21.3% identity with *S. aureus* NikA (E=2e-7), and 25.6% identity with *S. aureus* CntA (E=3e-28). Identical and similar amino acids are shaded black and gray, respectively, using BOXSHADE (http://www.ch.embnet.org/software/BOX_form.html). Dashes indicate gaps.

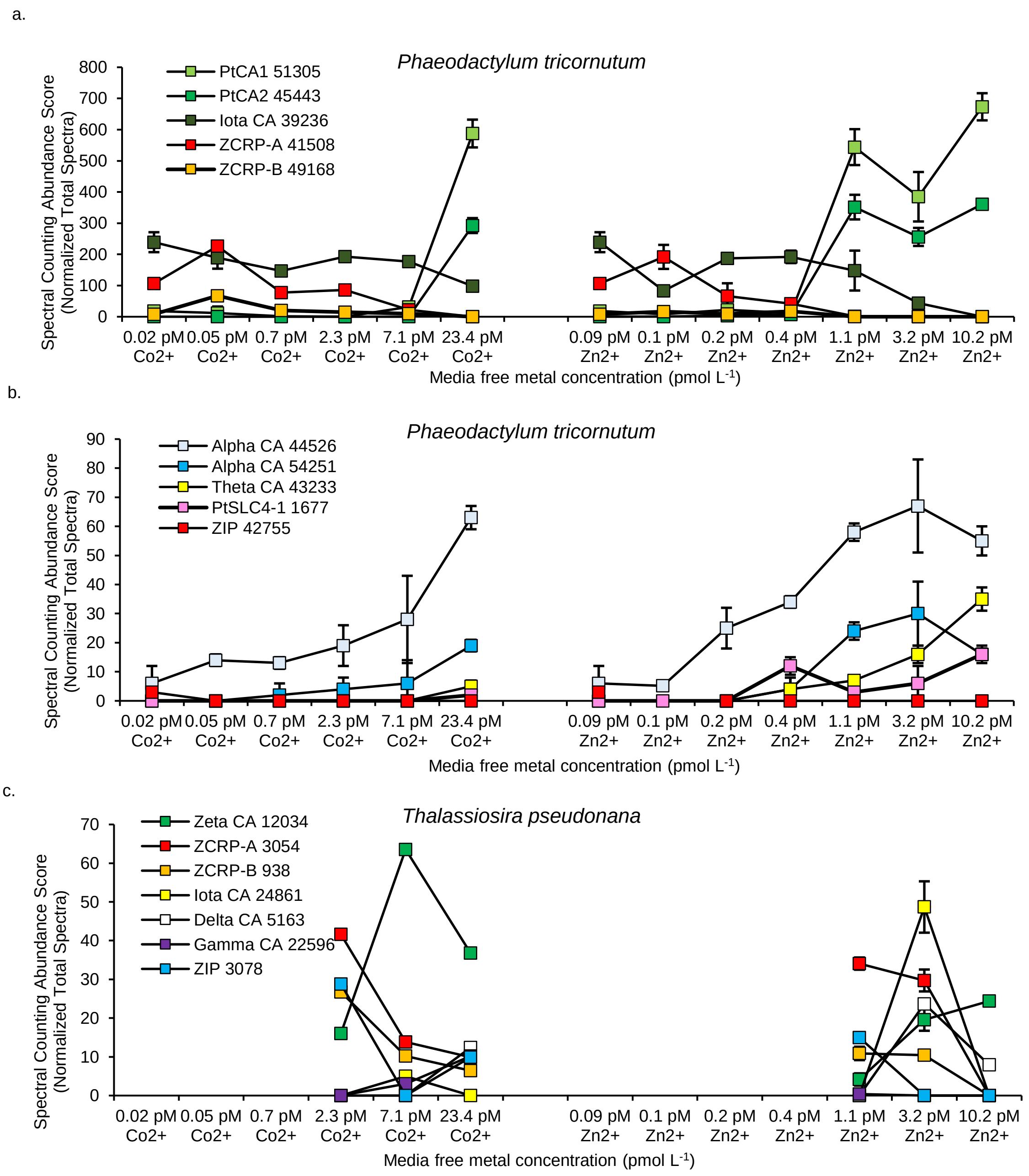

Supplementary Figure 7. Mean spectral counting abundance scores of all detected CAs and other proteins of interest in Zn and Co treatments of (a,b) *P. tricornutum* and (c) *T. pseudonana* as measured by global proteomic analysis. Data are presented as mean values ± the standard deviation of technical triplicate measurements of pooled biological duplicate cultures. *T. pseudonana* Co data are singlicate measurements. ZCRP-B abundances in (c) are plotted at a tenth of actual measured abundances for ease of trend comparison. Protein names are shown with their corresponding JGI protein ID. ND, no data.

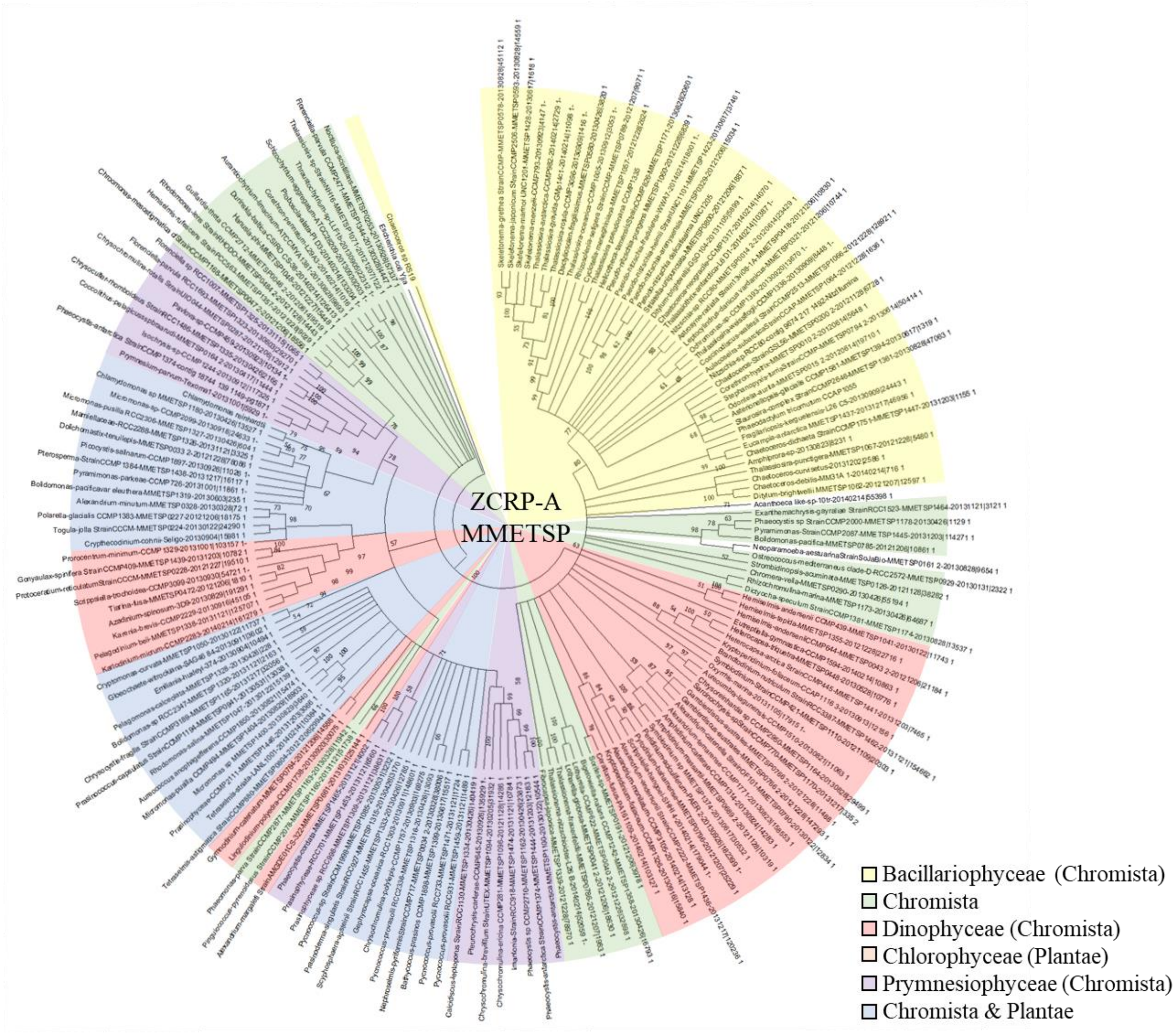

Supplementary Figure 8.
Phylogenetic tree of marine eukaryotic ZCRP-A homologs found in the MMETSP database. The evolutionary history was inferred using the UPGMA method [58]. The bootstrap consensus tree inferred from 1000 replicates is taken to represent the evolutionary history of the taxa analyzed [59]. Branches corresponding to partitions reproduced in less than 50% bootstrap replicates are collapsed. The percentage of replicate trees in which the associated taxa clustered together in the bootstrap test (1000 replicates) are shown next to the branches [59]. The evolutionary distances were computed using the Poisson correction method [72] and are in the units of the number of amino acid substitutions per site. This analysis involved 178 amino acid sequences. All ambiguous positions were removed for each sequence pair (pairwise deletion option). There were a total of 2718 positions in the final dataset. Evolutionary analyses were conducted in MEGA11 [57].

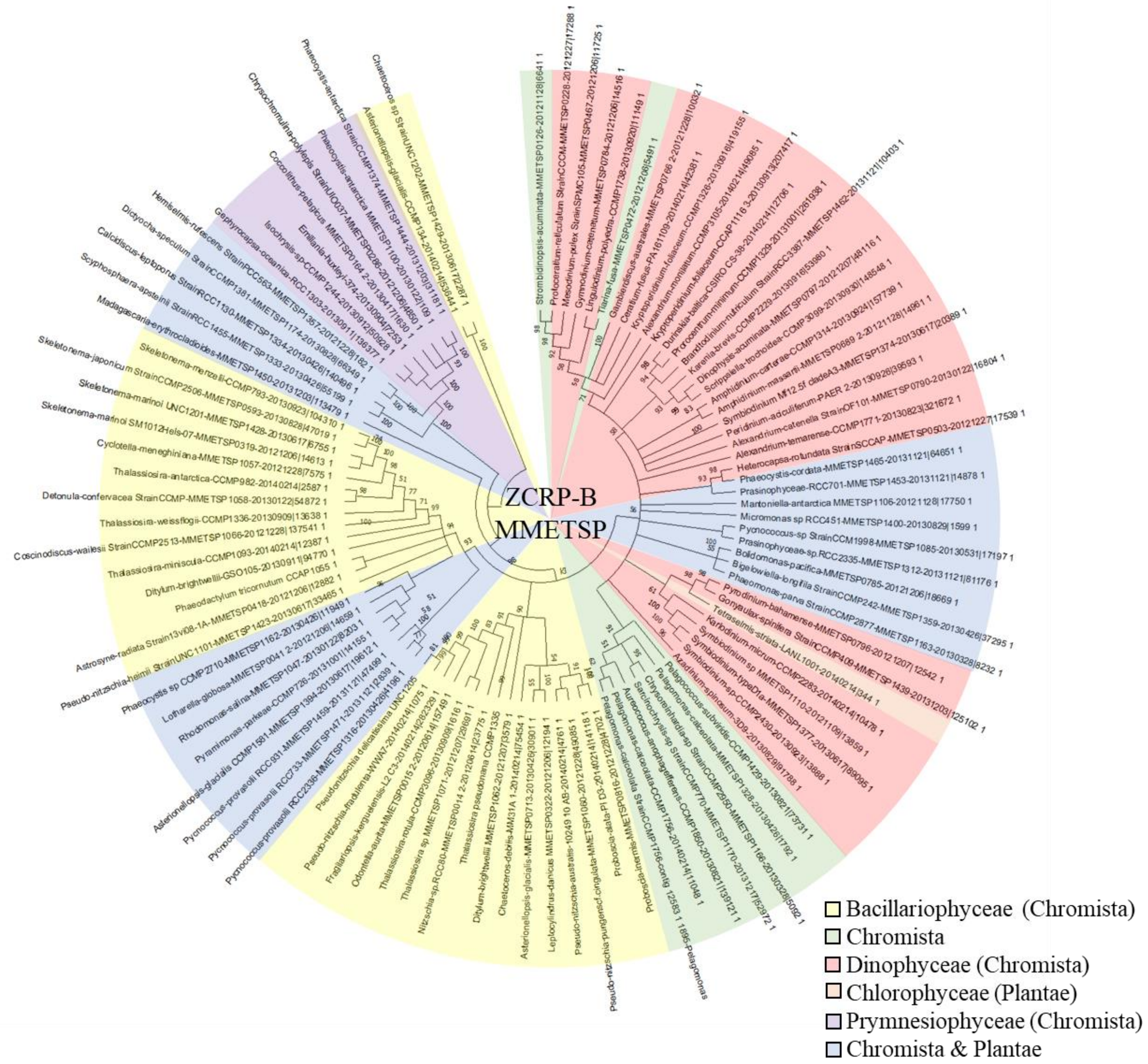

Supplementary Figure 9.
Phylogenetic tree of marine eukaryotic ZCRP-B homologs found in the MMETSP database. The evolutionary history was inferred using the UPGMA method [58]. The bootstrap consensus tree inferred from 1000 replicates is taken to represent the evolutionary history of the taxa analyzed [59]. Branches corresponding to partitions reproduced in less than 50% bootstrap replicates are collapsed. The percentage of replicate trees in which the associated taxa clustered together in the bootstrap test (1000 replicates) are shown next to the branches [59]. The evolutionary distances were computed using the Poisson correction method [72] and are in the units of the number of amino acid substitutions per site. This analysis involved 100 amino acid sequences. All ambiguous positions were removed for each sequence pair (pairwise deletion option). There were a total of 3196 positions in the final dataset. Evolutionary analyses were conducted in MEGA11 [57].

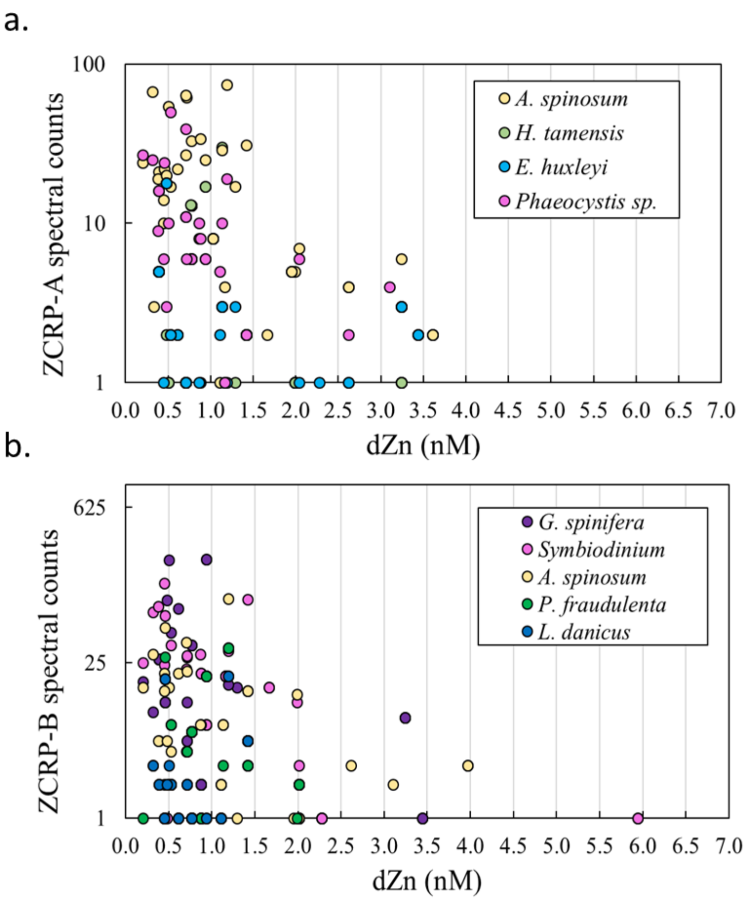

Supplementary Figure 10. Spectral counts of putative ZCRP-A and ZCRP-B homologs detected in the Pacific METZYME transect, represented on a log scale. (a) Spectral counts of ZCRP-A homologs detected in the dinoflagellate *A. spinosum*, the diatom *H. tamensis*, and the haptophytes *E. huxleyi*, and *Phaeocystis sp.* compared to dZn. (b) Spectral counts of ZCRP-B homologs detected in the dinoflagellates *G. spinifera*, *Symbiodinium sp.*, *A. spinosum*, and in the diatoms *P. fraudulenta* and *L. danicus* compared to dZn.
