# Supplementary material for: Adaptive responses of marine diatoms to zinc scarcity and ecological implications
Source: Nat Commun. 2022 Apr 14;13:1995. doi: 10.1038/s41467-022-29603-y (PMC9010474; doi:10.1038/s41467-022-29603-y)
Supplement: Supplementary file 3 — Description of Additional Supplementary Files [file 41467_2022_29603_MOESM3_ESM.pdf]

Description of Additional Supplementary Files

File Name: Supplementary Data 1
Description: Primer sequences
